# Correctly establishing evidence for cue combination via gains in sensory precision: Why the choice of comparator matters

**DOI:** 10.3758/s13428-023-02227-w

**Published:** 2023-09-20

**Authors:** Meike Scheller, Marko Nardini

**Affiliations:** Department of Psychology, Durham University, Durham, UK

**Keywords:** Cue combination, Sensory integration, Multisensory, Optimal observer model, Perceptual measurement, Psychophysics, Experimental design

## Abstract

Studying how sensory signals from different sources (sensory cues) are integrated within or across multiple senses allows us to better understand the perceptual computations that lie at the foundation of adaptive behaviour. As such, determining the presence of precision gains – the classic hallmark of cue combination – is important for characterising perceptual systems, their development and functioning in clinical conditions. However, empirically measuring precision gains to distinguish cue combination from alternative perceptual strategies requires careful methodological considerations. Here, we note that the majority of existing studies that tested for cue combination either omitted this important contrast, or used an analysis approach that, unknowingly, strongly inflated false positives. Using simulations, we demonstrate that this approach enhances the chances of finding significant cue combination effects in up to 100% of cases, even when cues are not combined. We establish how this error arises when the wrong cue comparator is chosen and recommend an alternative analysis that is easy to implement but has only been adopted by relatively few studies. By comparing combined-cue perceptual precision with the best single-cue precision, determined for each observer individually rather than at the group level, researchers can enhance the credibility of their reported effects. We also note that testing for deviations from optimal predictions alone is not sufficient to ascertain whether cues are combined. Taken together, to correctly test for perceptual precision gains, we advocate for a careful comparator selection and task design to ensure that cue combination is tested with maximum power, while reducing the inflation of false positives.

**Supplementary Information:**

The online version contains supplementary material available at 10.3758/s13428-023-02227-w.

Almost all environmental features can be perceived by means of multiple sensory signals that arise from different sources, also called sensory cues (see Table 1 for a list of frequently used terms). If two or more cues redundantly code for the same environmental feature, they can be integrated into the same perceptual representation. For instance, when determining the impact location of a bouncing ball, the observer can derive information about the location from both visual and auditory cues. Integrating these different sensory cues into a unified and coherent perceptual representation is a crucial process that allows humans to efficiently perceive and interact with their environment (Alais & Burr, 2019; Clark & Yuille, 1990; Ernst & Bülthoff, 2004; Landy et al., 1995; Stein et al., 2020; Wallace et al., 2020). An important feature that derives from the integration of multiple sensory cues is that the final, combined perceptual estimate is more precise than the perceptual estimates from each individual cue alone (Alais & Burr, 2019; Battaglia et al., 2003; Clark & Yuille, 1990; Ernst & Bülthoff, 2004). In other words, integrating information across multiple sensory modalities (or within sensory modalities) enhances perceptual precision.

**Table 1:** Description of frequently used terms

Term	Description
Cue	A sensory signal that arrives at our sensory receptors and contains information about its underlying source (environmental feature such as location, size, distance, weight, etc.)
Sensory noise $\sigma$	A measure that describes the uncertainty of a cue. Typically, this is estimated from the variability of the data distribution, or inverse slope of the psychometric function
Best cue $min({\sigma }_{1},{\sigma }_{2})$	Single cue with the lowest sensory noise (out of cue 1 and cue 2)
Worst cue $max({\sigma }_{1},{\sigma }_{2})$	Single cue with the highest sensory noise (out of cue 1 and cue 2)
Cue comparator	Single cue, for which the sensory noise is compared against that of both cues, to test for combination benefits
Group-determined best cue analysis ${\sigma }_{12} \mathrm{vs} {\sigma }_{1}$ *;* ${\sigma }_{12} \mathrm{vs} { \sigma }_{2}$	Sensory noise of the best (and worst) cue(s), selected at the level of the group, is compared with that of both cues. This is equivalent to comparing the raw individual cues to both cues (e.g., in an audio-visual paradigm: auditory vs audio-visual, visual vs audio-visual)
Individually-determined best cue analysis ${\sigma }_{12} \mathrm{vs} min({\sigma }_{1},{\sigma }_{2})$	Sensory noise of the best cue, selected at the level of the individual observer, is compared with that of both cues
Within-participant cue ratio *max(* ${\sigma }_{1},{\sigma }_{2}$*) / min(* ${\sigma }_{1},{\sigma }_{2}$*)*	Sensory noise of the worst cue over the sensory noise of the best cue, determined for each participant
Between-participant cue ratio proportion % ${\sigma }_{2}$ *<* ${\sigma }_{1}$	Proportion of participants for whom cue 1 has lower sensory noise than cue 2, determined at the group level
True combination effect	A statistically meaningful effect that truly reflects an increase in perceptual precision due to cue combination
False combination effect	A statistically meaningful effect that seems to reflect an increase in perceptual precision due to cue combination, but results from the inflation of false positives

Cue combination is nested in the processing hierarchy between low-level sensory processing and high-level conceptual representations. As a target of experimental investigation, it allows us to understand how we can gain a coherent percept of our environment from the complex and noisy signals that arrive at our senses at any moment in time. ‘*Noisy’* (or *sensory noise*) refers to the uncertainty that is inherent to all sensory signals and their neural encoding (Faisal et al., 2008), and is typically reflected in the variability of perceptual judgements. As such, studying cue combination provides a powerful approach to understanding perceptual processes as a form of probabilistic inference. A large body of research from the last two decades reported that probabilistic inference is consistent with common perceptual phenomena (e.g., Ernst & Banks, 2002; Knill & Saunders, 2003; Körding et al., 2007; Trommershäuser et al., 2012), illusions (Alais & Burr, 2004; Scheller et al., n.d.; Shams et al., 2005; Weiss et al., 2002), and allows to trace important perceptual differences between developmental or clinical groups (Bultitude & Petrini, 2021; Gori et al., 2008; Nardini et al., 2008; Nava et al., 2020; Negen et al., 2019; Petrini et al., 2014; Ramkhalawansingh et al., 2018; Scheller et al., 2020; Senna et al., 2021).

However, while methodological approaches to (behaviourally) quantify cue combination have been influenced by a small number of rigorous, psychophysical studies (e.g., Alais & Burr, 2004; Ernst & Banks, 2002; Hillis et al., 2004; see Rohde et al., 2016 for a tutorial), the last two decades have seen developments and diversification in procedures and analysis approaches. Most of them allow us to better understand different aspects of integration, to apply more careful approaches in differentiating integration from cognitive, perceptual, or design-induced biases, or to distinguish integration from alternative perceptual and cognitive mechanisms (Aston et al., 2022b; Ernst, 2012; Landy & Kojima, 2001; Moscatelli et al., 2012; Nardini et al., 2010; Otto et al., 2013; Rohde et al., 2016; Scarfe, 2022; Van Dam et al., 2014). At the same time, increasing popularity of the topic has led to the adoption of analyses that may not directly test one of the fundamental features of integration, that is, whether the combination of two cues leads to perceptually beneficial precision enhancement, relative to using either cue alone. In fact, the defining feature of cue combination – which most studies also state as the main reason for its investigation – is the enhancement of perceptual precision. As stated by Ernst & Bülthoff in their seminal work in 2004: “[…], the main purpose of sensory integration is to make the estimates more reliable. That is, there should be an observable reduction in variance compared with the individual estimates” (Ernst & Bülthoff, 2004, p. 165).

The present work argues that one of the most widely used criteria in testing for cue combination behaviour should be revisited, as its use suffers from an inflation of false positives, especially when certain design choices are not considered. Unfortunately, the analysis applied by the majority of studies that tested for cue combination falls into this category^1^. The present study further outlines under which conditions the inflation of false positives can occur, and how this pitfall can be avoided by following some simple steps.

First, this paper will introduce the concept of cue combination, outlining its most important experimental marker (a benefit in perceptual precision), and how this can be tested in a formalized way. It will also outline some of the other markers that researchers frequently test for, such as whether the magnitude of the benefit can be predicted by models of statistical optimality (see "Formalization and features of reliability-weighted/statistically optimal cue combination" section). We argue that such a test alone is not sufficient to evidence that two cues are indeed combined. Instead, comparisons have to be made between the individual cues and the combined cues. We further show how a researcher’s ability to measure cue combination depends on several participant-specific characteristics, such as the absolute and relative sensory noise levels of the individual cues. These determine the maximum possible benefit (i.e., maximum effect size) that an observer can obtain from combining sensory cues. As maximizing the possible benefit reduces the impact of measurement noise, we outline how taking these participant-specific characteristics into account when designing experiments can enhance our ability to empirically measure combination.

Next, we summarize different approaches that previous studies have employed to test for cue combination and evaluate the most commonly used methods, focusing on group-based rather than individual-observer analyses. In these approaches, researchers typically contrast the perceptual precision of observers when they are presented with two cues at the same time versus when they are presented with the individual, single cues. The cue comparator, that is the *individual cue* precision that is contrasted with the *combined cue* precision, differs between the methods that have been employed in the literature: the most common method uses the *group-determined best cue* as comparator, while the less common method uses the *individually determined best cue* as cue comparator. By generating data for an example experiment in which observers do not combine cues, we demonstrate the effect that the two different cue comparators have on measuring cue combination. We then show how the chances of finding *true* and *false combination effects* changes depending on the choice of cue comparator, as well as the maximum possible benefit. Lastly, by simulating data for an example standard cue combination experiment, we illustrate the degree of the problem that arises from using the wrong comparator, that is, the *group-determined best cue*. These simulations show that, if choosing this comparator, our chances of finding false positives increases up to 100%. Instead, when using the *individually determined best single* cue as comparator, false-positive rates are kept below the generally accepted 5% rate.

## Formalization and features of reliability-weighted cue combination

Cue combination studies compare perceptual precision of two cues (e.g., an auditory and a visual cue to a target’s location) presented together with the perceptual precision of either cue on its own. Placing cue combinations within the framework of statistically optimal integration, the magnitude of perceptual benefits when given both cues together vs either alone in well-controlled laboratory experiments is often consistent with a weighted linear combination of the two cues (Alais & Burr, 2004; Ernst & Banks, 2002; Hillis et al., 2004). Formally expressed, when perceiving an object feature via redundant information, each cue (*i* = 1, 2, …, *n*) can be represented as an independent, sensory estimate (${\mu }_{1}$, ${\mu }_{2}$, …, ${\mu }_{n}$) of the external stimulus property (*X*) that is corrupted by sensory noise (${\sigma }_{1},{\sigma }_{2},\dots ,{\sigma }_{n}$), such that ${\mu }_{i} \sim N(X,{{\sigma }_{i}}^{2})$.

The noise of a cue can be taken as a measure of sensory uncertainty during probabilistic perceptual processes. The inverse of a cue’s noise is expressed as its reliability *rel*, i.e., ${rel}_{i}= {{\sigma }_{i}}^{-2}$. In most cases, researchers can assume that the noise is normally distributed and is not correlated across cues (Ernst, 2007; Rohde et al., 2016) although this may not always be the case (Ernst, 2012; Oruç et al., 2003). Under these assumptions, the combination of two cues that are weighed by their individual reliabilities, $\omega ={rel}_{i}/{\sum }_{i}{rel}_{i}$, would lead to reductions in sensory noise in line with maximum likelihood estimation (MLE). Hence, the smallest possible sensory noise that can be achieved via reliability-weighted integration, ${\sigma }_{12,mle}$, is given by:

1 $${\sigma }_{12,mle}= \sqrt{\frac{{\sigma }_{1}^{2} \cdot {\sigma }_{2}^{2}}{{\sigma }_{1}^{2} + {\sigma }_{2}^{2}}}$$

**Fig. 1:**
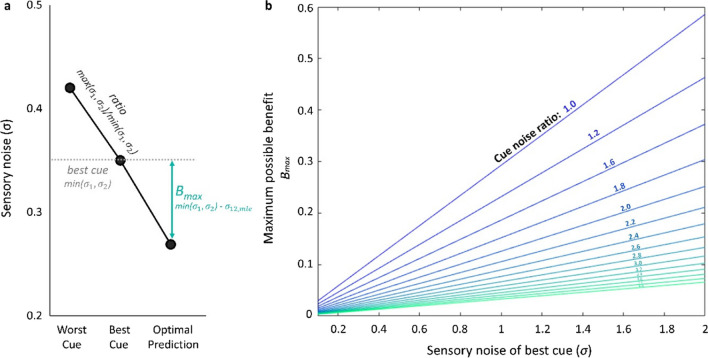
**a** The maximum possible benefit (*B*_max_) that the perceptual system can achieve by combining two redundant cues in a reliability-weighted fashion. The plot shows how the maximum benefit is derived from the sensory noise level difference between the best sensory cue, *min(*${\sigma }_{1},{\sigma }_{2}$*),* and the optimal prediction, ${\sigma }_{12,mle}$ (Eq. 1). **b** As the maximum benefit follows from the sensory noise values of both individual cues (${\sigma }_{12,mle}$) its magnitude depends on the absolute sensory noise in the best single cue, as well as the sensory noise ratios of both single cues. Increasing sensory noise in the best cue and matched cue ratios lead to a larger possible benefit

As this optimal estimate takes the single-cue reliabilities into account, the maximum possible benefit that an observer can gain by integrating two cues by their relative reliabilities (and hence, the maximum possible benefit that a researcher can expect to measure: ${B}_{\mathrm{max}}={\sigma }_{\mathrm{best}}- {\sigma }_{12,mle}$^2^) is influenced by the absolute sensory noise of the best single cue, as well as the sensory noise ratio between the two single cues (ratio = *max(*${\sigma }_{1},{\sigma }_{2}$*)* / *min(*${\sigma }_{1},{\sigma }_{2}$*)*; see Fig. 1).

Larger sensory noise values in the individual cue conditions can lead to a larger potential benefit, in line with the inverse effectiveness principle, which has been frequently evidenced in studies on the neural mechanisms underlying multisensory integration as well as behaviour (Frassinetti et al., 2002; Hecht et al., 2008; Meredith & Stein, 1986; Møller et al., 2018; Stein et al., 1988, 1989, 2009; Stevenson et al., 2012). That is, the enhancement in neural responses and perceptual precision that are obtained from combining two cues is larger when uncertainty in the two single cues is high and more similar. Hence, in order to allow for a larger benefit and therefore possible effect size, researchers might be inclined to design experiments in which individual cue noise is high.

However, aiming to attain very large sensory noise values can pose serious issues for measuring cue combination. For instance, as large sensory noise values translate into impoverished stimulus representations and low stimulus discriminability, they necessitate making perception more difficult by means of decreasing stimulus reliability (for instance by selecting a narrower stimulus range). Practically implemented, this can lead to demotivation in participants, decreases in attention, and lower data quality. At the same time, if sensory noise is extracted from modelling the task data, such as with two-alternative forced choice (2AFC) response tasks, and responses do not plateau at extreme stimulus levels, this complicates parameter estimation by leading to lower differentiability of sensory noise and lapses (nuisance related to noise that is tangential to the decision; Prins, 2012; Wichmann & Hill, 2001). Overall, higher sensory noise values are more difficult to recover as they are less distinguishable from lapses (more details in Supplementary Materials). Hence, we do not recommend that researchers aim to increase the sensory noise in the best single cue to enhance their ability of measuring cue combination effects. Instead, the cue noise ratio of the individual cues should be considered.

Indeed, the maximum possible reduction in uncertainty is not only affected by the best cue’s absolute sensory noise, but also by the relative reliabilities of the two cues, that is, the uncertainty ratio of the worst to the best cues (henceforth: *within-participant cue ratio*). This is an important consideration for cue combination assessments and has also been clearly outlined in previous work (Scarfe, 2022). While well-matched cues (within-participant cue ratio = 1) allow for larger reductions in uncertainty, an increase in the ratio markedly reduces the possible benefit that can be measured. In some instances, such as when individual cue reliabilities are not well matched, optimal predictions cannot be distinguished from the best single cue (e.g., de Winkel et al., 2010). This is because the maximum possible benefit can become even smaller than the measurement error (e.g., parameter estimation uncertainty). Hence, when within-participant cue ratios are high it becomes more difficult to determine whether the nervous system truly implements statistically optimal integration, or whether the less precise single cue is discounted and the more precise single cue is followed (see also Scarfe, 2022).

Which cue is most informative can further differ between individual observers. Due to large inter-individual differences in sensory reliabilities, it is challenging to anticipate both the best cue noise levels, and the *within-participant cue ratios* for a group of participants. However, Fig. 1b demonstrates how much the possible benefit (i.e., the largest possible effect size) depends on those participant-specific characteristics. This not only makes sample size and power estimation difficult but also emphasizes that most cue combination studies are dealing with very small (maximum possible) effect sizes. Single studies have often attempted to achieve higher power either (1) by minimizing measurement noise through robust designs with many repetitions and individual threshold-calibrations in small samples using individual observer analyses^3^ (*n* ≤ 8; e.g., Alais & Burr, 2004; Ernst & Banks, 2002; Rosas et al., 2005) or (2) by testing larger, more representative samples of individuals and applying group-level analysis (e.g., Adams, 2016; Gori et al., 2008; Helbig & Ernst, 2007, 2008; Jicol et al., 2020; Meijer et al., 2019; Nardini et al., 2008; Newman & McNamara, 2021; Plaisier et al., 2014; Zhao & Warren, 2015). However, a priori power estimation has rarely been conducted in cue combination studies (see also Scarfe, 2022), typically because these participant-specific characteristics are difficult to gauge if they are not individually calibrated in advance (but see Meijer et al., 2019).

## Different approaches to quantifying cue combination

Over the years, multiple different ways of analysing and quantifying cue combination have been employed. While the most frequently used analyses were conducted at the group level, a small number of early but influential studies conducted individual-level analyses, typically with smaller samples being tested. In some cases, more than one analysis, or additional visualization strategies were used to evidence integration. A summary of these previously employed approaches is outlined below.^4^

- The most common way in which cue combination has been evidenced in previous studies is through contrasting sensory noise of the combined cue condition with that of the individual, single cues (separated by cue type). For example, in a visuo-haptic paradigm where ${\sigma }_{1}$ denotes the sensory noise of the visual cue and ${\sigma }_{2}$ denotes the sensory noise of the haptic cue, Helbig and Ernst (2007) compared the sensory noise levels of the visuo-haptic combined condition ${\sigma }_{12}$ with the single-cue visual condition and the single-cue haptic condition. This contrast is given by:
  2 $${\sigma }_{12}\; \mathrm{vs }\; {\sigma }_{1} \;;\; {\sigma }_{12}\; \mathrm{vs }\; {\sigma }_{2}$$
  By splitting the single-cue comparators by their cue type, data from observers with higher precision in cue type 1 compared to cue type 2, and vice versa, are mixed. Hence, the main comparators that bimodal performance is contrasted with are *the ‘group-determined best’ and ‘group-determined worst’ cues*. Sometimes, only the group-determined best cue is used as comparator, as significant effects relative to this cue can make the contrast with the group-determined worst cue redundant. The vast majority of studies that tested for cue combination used this approach (e.g., Adams, 2016; Bates & Wolbers, 2014; Bultitude & Petrini, 2021; Burr et al., 2009; Chancel et al., 2016; Chen et al., 2017; Elliott et al., 2010; Ernst & Banks, 2002; Fetsch et al., 2009; Frissen et al., 2011; Gabriel et al., 2022; Gibo et al., 2017; Goeke et al., 2016; Gori et al., 2008, 2021; Gori et al., 2012a, b; Helbig & Ernst, 2007, 2008; Jicol et al., 2020; Jürgens & Becker, 2006; MacNeilage et al., 2007; Nardini et al., 2008, 2010; Newman & McNamara, 2021, 2022; Petrini et al., 2014, 2016; Ramkhalawansingh et al., 2018; Risso et al., 2020; Scheller et al., 2020; Seminati et al., 2022; Senna et al., 2021; Sjolund et al., 2018; Zanchi et al., 2022; Zhao & Warren, 2015).
- Another way in which cue combination has been evidenced at the group level is by contrasting the combined cue condition with the *individually determined best cue*. Here, an additional step is implemented in the analysis that determines, for each observer, which of the two individual cues is less noisy. This less noisy (i.e., individually determined best) cue is then used as a comparator in group analyses to test for benefits in precision:
  3 $${\sigma }_{12}\;\mathrm{ vs\; min}({\sigma }_{1},{\sigma }_{2})$$
  However, while this additional step is necessary to truly test for precision benefits in perception at the group level, a much smaller number of studies has employed this approach (Alais & Burr, 2004; Arnold et al., 2019; Aston et al., 2022a; Ball et al., 2017; Butler et al., 2010; Garcia et al., 2017; Negen et al., 2018, 2019; Plaisier et al., 2014).
- Additionally, alongside employing one of the above analysis, perceptual benefits are frequently tested for optimality. That is, the sensory noise of the combined condition is contrasted with the lowest possible sensory noise, which is obtained from MLE predictions.
  4 $${\sigma }_{12}\; \mathrm{vs }\; {\sigma }_{12,mle}$$
  As the predicted optimal performance provides a useful minimum possible comparator that is scaled by the individual cue noise values, it makes it possible to test whether any benefit shown in the previous analysis also meets the predictions of statistical optimality (Rohde et al., 2016). In other words, it accounts for the fact that some individuals may only obtain a small benefit from combining two cues, such as when sensory noise ratios are high, while other individuals can gain a larger benefit. A number of more recent studies made use of this prediction and quantified the benefit of cue combination through the difference in sensory noise between the combined cue condition and the MLE predictions (Heffer et al., 2022; Nava et al., 2020; Scheller et al., 2020; Senna et al., 2021):
  5 $$Combination\; index= {\sigma }_{12}- {\sigma }_{12,mle}$$
  As most of these studies investigated the effects of (sub-)clinical conditions or development on multisensory integration, this difference score provided a useful approximation of the degree of integration, relative to the maximum benefit, that could then be contrasted between groups. However, it should be noted that reporting this score or contrast with the MLE prediction alone (e.g., Nava et al., 2020; Takahashi et al., 2009; Takahashi & Watt, 2017) does not provide evidence that two cues were indeed combined. In other words, it is unclear whether the groups differed in integration, or changes in the maximum possible benefit. Without contrasting the empirically measured bimodal sensory noise levels with single-cue sensory noise levels, perceptual benefits that exceed the best single-cue performance cannot be evidenced, and it cannot be ascertained that cues were combined. Therefore, such combination indices should only be used in addition (e.g., as in Heffer et al., 2022; Scheller et al., 2020; Senna et al., 2021) but not instead of the crucial analysis that tests for cue combination.
- Some further studies, especially those that included small samples (*N* ≤ 8) as a result of more complex designs (e.g., multiple levels of conflict and noise manipulations, multiple sessions, rare patient groups or slow presentation options) based their conclusions on comparisons at the individual observer level (de Winkel et al., 2013; Oruç et al., 2003; Risso et al., 2019; Rosas et al., 2005) which often included bootstrapping, or even purely visual/descriptive approaches^5^. While this allows inferences about integration benefits (based on individuals’ comparisons between the best and combined cues), it can still be problematic: given that the possible benefit that can be gained from optimal integration is rather small, this approach often lacks the statistical power to detect such small benefits. This is especially true when individual measures derive from little data and parameter estimates are affected by measurement noise that is larger than the possibly obtainable benefit. Notably, measurement noise is often not quantified or accounted for, but can be partially averaged out by employing a group-based approach. Nevertheless, testing large groups of participants with complex designs is not always feasible to address certain questions. Hence, careful design, such as calibrating single cues (to increase the possible benefit) or increasing the number of stimulus repetitions for each stimulus level (to decrease measurement noise) can improve small sample studies that rely on individual-based comparisons.
- Some cue combination studies employed more than one approach, and complemented group- based statistical analyses with additional, observer-based visualizations or descriptives (Kaliuzhna et al., 2015; Meijer et al., 2019; Nardini et al., 2013; Petrini et al., 2014; Rosas et al., 2005; Scheller et al., 2020). Providing such additional evidence is useful in that it allows to determine whether integration was beneficial for a certain proportion or sub-group of observers within the whole sample. However, making judgements about the combination of cues based on visual and descriptive comparisons alone is highly problematic (see also Scarfe, 2022), and should therefore only be used as complementing information, but not sole evidence for cue combination.

## Present study

In previous studies, the rationale for choosing a specific analysis approach has rarely been explicitly stated. Are these approaches equally powerful in determining true cue combination effects? Crucially, most studies state that they test for cue combination because it benefits perception by reducing sensory noise in the combined estimates. We therefore argue that in order to evidence true cue combination, the crucial comparison should not be limited to whether bimodal noise levels differ from optimal predictions, but, more fundamentally, whether bimodal noise levels are reduced (improved) relative to the noise levels of single cues.

Furthermore, by acknowledging that perception is a process that takes place within, rather than across individuals, it becomes evident that the reference cue against which bimodal noise levels should be compared is not determined at the group level, but instead at the level of the individual participant (Grice et al., 2017; Smith & Little, 2018). Therefore, the critical test for cue combination at group level is whether the measured bimodal noise levels are lower than that of the observers’ best single-cue noise levels. By employing group analyses that use the group-determined best single-cue noises as comparators, many researchers have unknowingly enhanced the occurrence of false positives in their research design. The following example scenario demonstrates how this can happen.

## Effects of the different cue contrasts

Suppose we are interested in finding whether two cues are combined to perceive the depth of an object in space. For each of the two cues, as well as the combined condition, we collect repeated depth judgements in a 2AFC paradigm and derive sensory noise values (discrimination thresholds/just-noticeable-differences/response variability) for 18 naïve observers. This is around the average number of participants that is included in many cue combination studies (e.g., Chancel et al., 2016; Goeke et al., 2016; Nardini et al., 2008; Petrini et al., 2016; Ramkhalawansingh et al., 2018). Let us further suppose that for five of these participants cue 1 is more precise than cue 2, while for the remaining 13 participants cue 2 is more precise. That means, the *between-participant cue ratio proportion* is 72% ${\sigma }_{2}$ < ${\sigma }_{1}$. There is large variability in the literature in the between-participant cue ratio proportion, and most studies do not even report this measure. However, when attempting to match the individual cue reliabilities (as we recommend above, and has been recommended by Rohde et al., 2016 and Scarfe, 2022) it can be expected that the proportion of participants for whom cue 2 is more precise than cue 1 approaches an even split of around 50%. This is an important factor to bear in mind for the choice of analysis, as we will outline below. For demonstration purposes, let the *within-participant cue ratio* of the worst to best cue be 3 for all individuals. Again, this is a parameter that strongly affects our ability to find cue combination but is typically not reported in the literature. Lastly, in our example, the combined cue sensory noise was drawn from a normal distribution centred on the best sensory cue, with a SD of 0.02, which can be expected from measurement noise alone. In other words, on average, participants followed the best sensory cue (they did not integrate the cues), but there was a small degree of variation at the individual level.

**Fig. 2:**
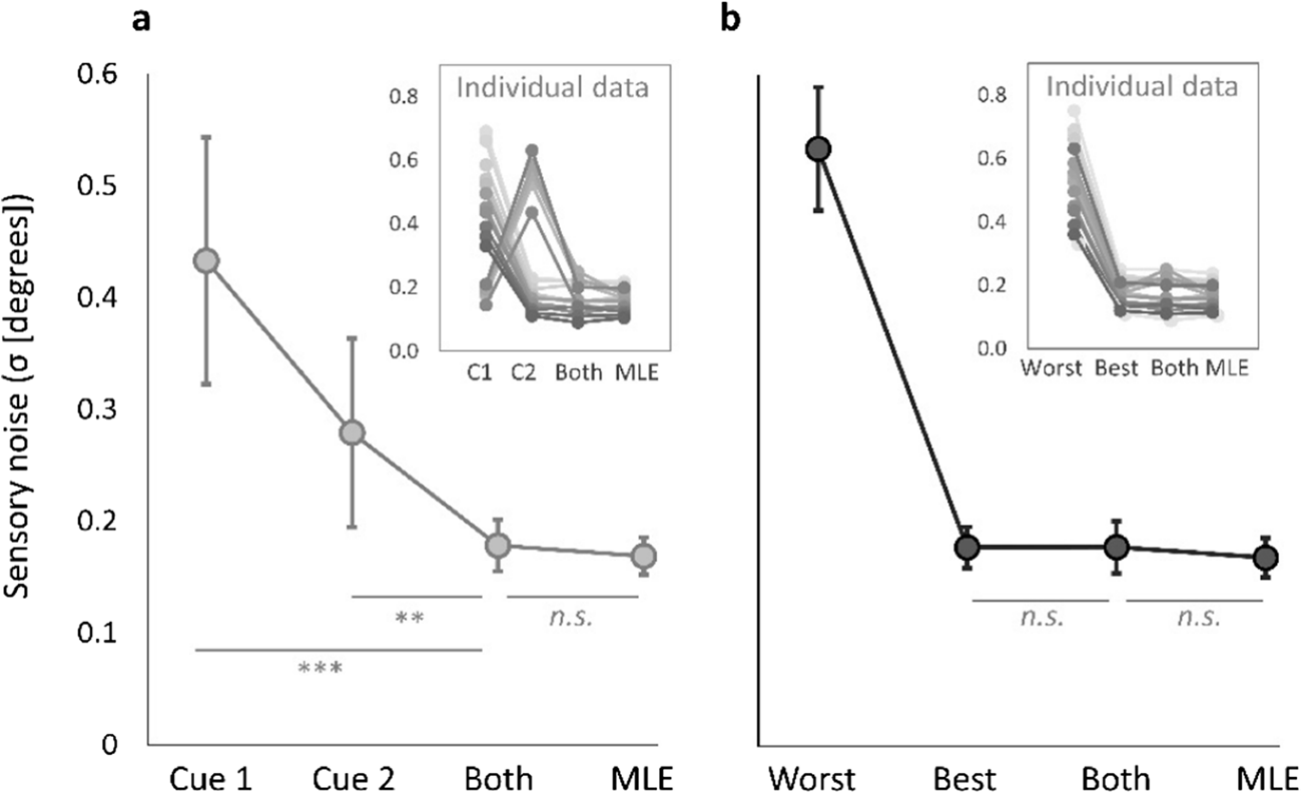
Visual demonstration of the effects of the two analysis methods. Left and right panels plot the same sensory noise values for a simulated experiment with 18 observers (see main text for details). Larger panels show the sensory noise values averaged across the group, while smaller inlets show the data of the individual observers. The difference between panels **a** and **b** is the split of the single-cue conditions, which form the cue comparators for the combined condition (both): panel **a** indicates the more common analysis whereby the combined cue condition is contrasted with the group-determined worst and *group-determined best single cues* (similar to splitting them by sensory modality, e.g., visual, haptic). Panel **b** indicates the less common, but correct, analysis, whereby the combined cue condition is contrasted with the *individually determined best sensory cue*. *Error bars* indicate 95% confidence intervals. Despite using the same data, the results we obtain when testing for precision benefits differ between the analyses shown in panels **a** and **b**: Paired signed-rank tests indicate significant improvements for paired cue conditions when compared with the group-determined best and worst cues (panel a: Cue 1 vs Both: *p* = 0.002; Cue 2 vs Both: *p* = 0.003; *p* values are Holm–Bonferroni-corrected), but not when compared with the *individually determined best cue* (**b** Best vs Both: *p* = 0.388). In panel **a**, this indicates a *false combination effect*, resulting from the inflation of sensory noise levels in cue 2, leading us to the erroneous conclusion that combination effects are present in this data, when they are not. Note that, in both cases the combined cue noise does not differ from MLE predictions. While the true possible benefit that can be obtained from optimal combination is very small in both cases (*B*_max_* = *MLE - best cue; Here, *B*_max_* = *0.01), averaging across sensory noise values before selecting the best and worst cues for each observer reduces the apparent sensory noise ratio of the single cues and thereby exaggerates the apparent magnitude *B*_max_

**Fig. 3:**
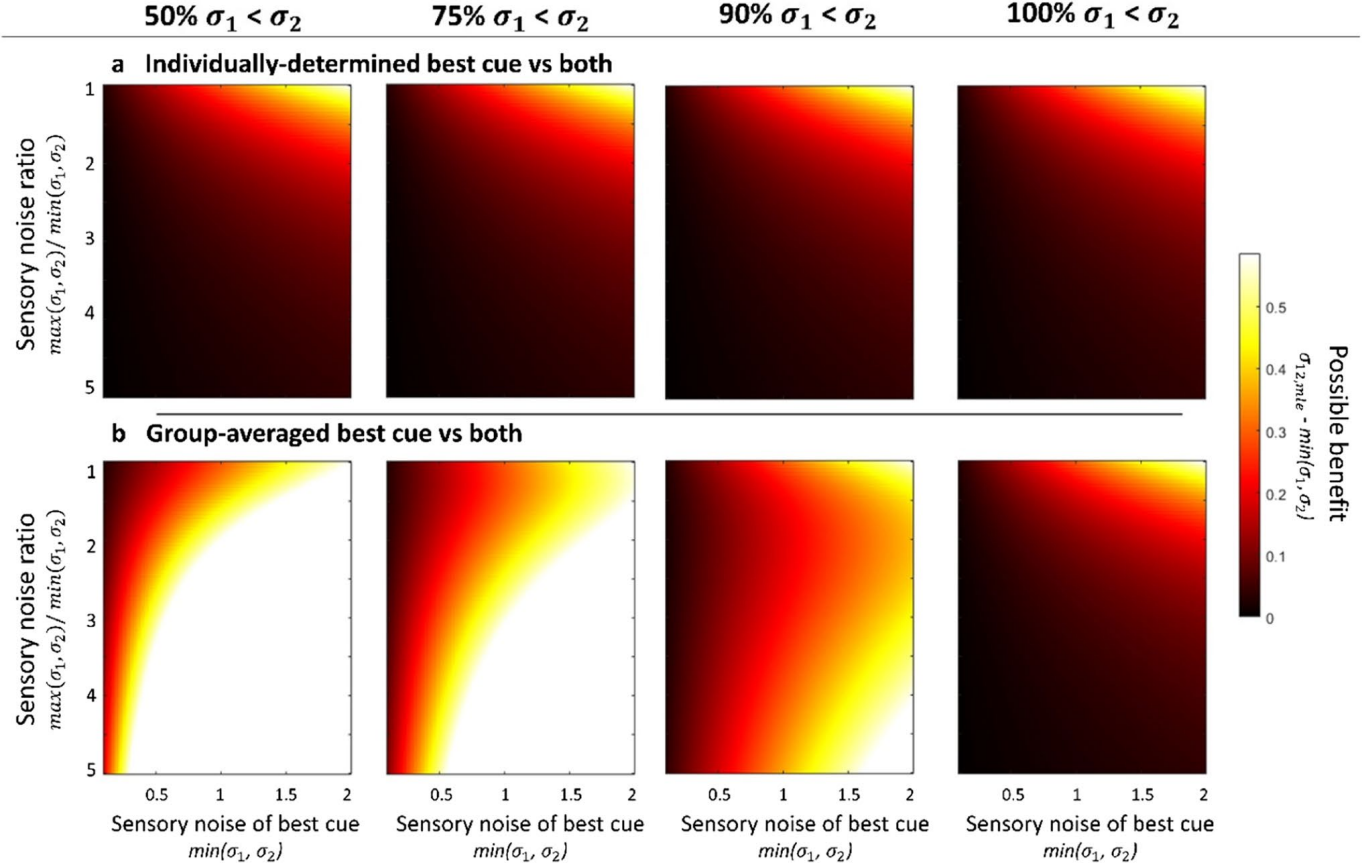
Heatmaps showing how the maximum possible benefit (*B*_max_) depends on the sensory noise of the best cue, *min(*${\sigma }_{1},{\sigma }_{2}$*)*, sensory noise ratio, *max(*${\sigma }_{1},{\sigma }_{2}$*)/min(*${\sigma }_{1},{\sigma }_{2}$*)*, the proportion of participants for which one of the two cues is more precise than the other one, i.e., *x% *${\sigma }_{1}$*<* ${\sigma }_{2}$, as well as the comparator that is chosen for the analysis. **a** By contrasting sensory noise values of the *individually determined best cue* with the combined cue condition, i.e., *min(*${\sigma }_{1},{\sigma }_{2}$*)* vs ${\sigma }_{12}$, the possible benefit remains constant, independently of the proportion of participants for which cue 1 is more precise than cue 2 (*panels left to right are the same*). This analysis tests for a true combination effect. **b** On the contrary, when the *group-determined best cue* noise is contrasted with the combined cue noise, i.e., *min(*$\widehat{{\sigma }_{1}},\widehat{{\sigma }_{2}}$*)* vs ${\sigma }_{12}$, the maximum possible benefit is enhanced. This enhancement does not, however, reflect *true combination* but rather increases the difference between MLE prediction (which stays constant) and the comparator (*group-determined best cue*) by inflating sensory noise values in the latter. The effect is stronger when the population of individuals having cue 1 vs 2 as their best single cue is more mixed (*panels towards the left*)

In order to assess the evidence for cue combination, we are now interested in testing whether noise levels are reduced in the bimodal cue condition. However, depending on the single-cue condition that is used as comparator (section 3a vs section 3b), the outcome of our analysis differs starkly. Figure 2 illustrates this visually. It shows the same sensory noise values for each cue condition plotted either with the *group-determined best and worst cues* (i.e., section 3a, Fig. 2a) or with the *individually determined best and worst cues* (section 3b, Fig. 2b). By contrasting sensory noise of the combined cue condition with that of *group-determined best and worst cues* (or even just the *group-determined best cue*, i.e., cue 2 in Fig. 2a), the higher sensory noise value in the comparator suggests that there is an appreciable benefit in the combined condition. However, when looking at the individual sensory noise values (smaller figure within the same panel), it becomes clear that the suggestive benefit results only from an averaging-induced increase in sensory noise levels of the cue comparator: cue 2. Furthermore, due to the large *within-participant cue ratio*, which appears to be reduced by averaging over individuals, the maximum possible benefit appears larger in the left panel. However, the actual maximum possible benefit remains very small, as can be seen in the individual observer plot as well as the right panel (Fig. 3b).

By contrasting the combined condition with the *group-determined best cue*, we observe a significant decrease in sensory noise in the combined condition (Fig. 2b). We call this false positive a *false combination effect*. It describes a significant reduction in sensory noise when both cues are available, compared to the individual single cues, resulting from an inflation of the single-cue noise levels rather than a true noise reduction (precision increase) in perception. This false combination effect remains significant even after adjusting for multiple comparisons. Hence, adopting this analysis approach would lead us to conclude that the participants in our example experiment gain precision by combining both cues in a near-optimal fashion, even though there is no *true combination effect* in the data. A *true combination effect* is described as a significant reduction in sensory noise when both cues are presented together, compared to the best single cue, as a result of a real increase in perceptual precision.

By contrasting the sensory noise of the combined cue condition with the best single cue, selected for each participant individually, we find that there is no significant reduction in sensory noise, and hence, no precision enhancement. This accurately reflects the true negative that is given by our example. We further see that the minimal possible benefit in precision (indicated by the best vs MLE predicted noise values; average *B*_max_* = *0.009) that results from the high sensory noise ratio between the two individual cues makes it very difficult to distinguish ‘optimal combination’ from ‘no combination’. This would be particularly problematic in a real data set in which *true combination* could potentially occur – however, as we have knowledge about the underlying distributions in our example data, we can be certain that we should not find any systematic precision improvement.

Crucially, the individual observers’ perceptual characteristics (e.g., the absolute cue noise levels) affect not only how large the maximum benefit is that can be obtained from optimal combination, but therefore also the degree of alpha error inflation when the *group-determined best (and worst) single cue(s)* is chosen as comparator. That is, as observers differ in their perceptual abilities, some participants would naturally end up with one cue being better than the other. The proportion of observers that show lower sensory noise levels in one cue compared to the other cue (henceforth: between-participant cue ratio proportion) determines whether we are more likely to find a true or false combination effect. To investigate further how the expected alpha error changes as a function of this between-participant cue ratio proportion in the sample, we calculated the maximum possible benefit (*B*_max_) an ideal observer can obtain, under different proportions. As a larger *B*_max_ magnitude decreases the relative influence of measurement noise (assuming measurement noise stays constant), it enhances the chances of finding (true and false) combination effects. Furthermore, as outlined in section 1.2, the magnitude of *B*_max_ is largest for high sensory noise values in the single cues and for low within-participant cue ratios.

Importantly, the maximum possible benefit is not affected by the proportion of observers for whom one specific cue is the more precise than the other one (i.e., the between-participant cue ratio proportion) when the comparator in the analysis is *the individually determined best single cue* (Eq. 3; Fig. 3, top row). However, when the comparator in the analysis is the *group-determined best single cue* (equivalent to contrasting cue 2 and both cues in our example above; Eq. 2), the possible benefit *B*_max_ appears to be larger (Fig. 3, middle row). This increase in *B*_max_ is particularly large when within-participant sensory noise ratios are high (lower in each panel) and when the between-participant cue ratio is more evenly split (left panels). Notably, as this enhancement stems from an increase in the sensory noise levels of the individual cue comparator (by combining the worse and best cues of different participants), it does not only affect *B*_max_, but also the contrast of interest, that is, the combined cues versus single cue noise levels.

If the between-participant cue ratio proportion is evenly split within the sample (i.e., 50% ${\sigma }_{1}$*<* ${\sigma }_{2}$), the inflation of false positives increases. In contrast, if one cue is relatively more precise than the other for the whole sample (e.g., 100% ${\sigma }_{1}$*<* ${\sigma }_{2}$), there is no inflation of false positives. However, such a scenario is typically more likely to occur when one of the cues is considerably more precise than the other, likely resulting in high within-participant cue ratios, which, in turn, reduce the chances to detect true combination effects. Hence, when reducing the noise ratios of the single cues for all individual observers, it is more likely to end up with a more evenly split between-participant cue ratio proportion (i.e., more like 50% ${\sigma }_{1}$*<* ${\sigma }_{2}$).

## How cue comparator choice leads to false and true combination effects ‐ a simulation example

To test the effects that the two different analysis approaches have on the chances of obtaining a true or a false combination effect, we simulated data for a hypothetical cue combination experiment under a range of conditions. A similar approach has recently been introduced by Scarfe (2022). Here, we directly contrasted the outcomes of the two methods, ‘using the group-average best cue as cue comparator’ (section 3a) and ‘using the individually selected best cue as cue comparator’ (section 3b), with simulated data from observers who either combined the cues in line with predictions of statistical optimality (Eq. 1) or who did not combine the cues but followed the best sensory cue while ignoring the worse cue (*min(*${\sigma }_{1},{\sigma }_{2}$*)* = ${\sigma }_{12}$).

 We simulated responses for a feature discrimination task that used a 2AFC paradigm with a sampling method of constant stimuli, which has frequently been used by many psychophysical cue combination studies (Ernst & Banks, 2002; Kingdom & Prins, 2016; Rohde et al., 2016). Simulated observers were tasked with determining which of two consecutively presented objects had a greater magnitude, specifically, which one was larger in size. The stimulus feature range was log-transformed and, for comparability, normalized such that all values fell between – 1 (e.g., smaller) and 1 (e.g., bigger). Based on 20 repetitions for each of 14 comparison stimulus levels, we generated responses of the target being reported to be larger than the reference, for each cue condition (cue 1, cue 2, both) and each observer.

As can be expected with human participants, simulated observers exhibited lapses, which randomly affected between 1% and a maximum of 10% of trials. While lapses affect performance, they often lie outside of the experimenter’s control, and can be influenced by many factors that impact the observer’s ability to focus on the task (e.g., difficulties focussing on the task, confusing response keys, lack of rest or increasing fatigue from long sessions). While lower lapse rates (1–3%) can be expected in well-behaved, focussed participants, additional factors such as dual tasks, very long or tiring tasks, or inclusion of specific clinical or developmental populations can bring about increases in lapses. While it is difficult to control or directly assess the lapse frequency, researchers cannot assume that observers’ performance is free from these effects, and it is important to factor such human error into the response when simulating observers.

**Fig. 4:**
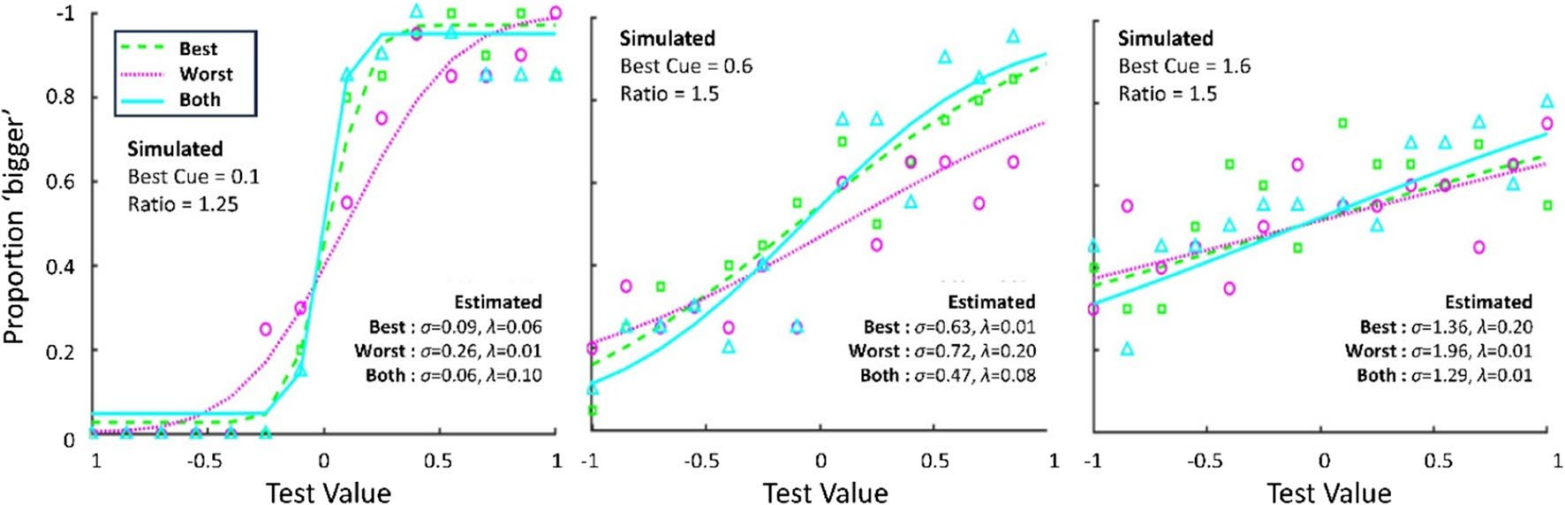
Example data and fitted psychometric functions of three simulated observers that combined cues according to Eq. (1). *Different colours and line types* represent the three different cue conditions (best single cue, worst single cue, combined cues). Simulated best cue noise levels and ratios of single cues are indicated left in each figure. Estimated sensory noise and lapse rate parameters for every cue are given on the right of each figure. All three observers differed in their participant-specific characteristics, with increasing levels of sensory noise of the best cue and sensory noise ratios from left to right. These are split across different panels in Fig. 5

A psychometric function of the form

6 $$\Psi \left(x; \mu , \sigma , \lambda \right)= \left(1-\lambda \right)*F(x| \mu ,\sigma )$$

was fit to the simulated proportions of responses stating that the stimulus feature was larger in magnitude (e.g., bigger size; Fig. 4). Here, $\lambda$ refers to the lapse rate, which was free to vary between 0.01 and 0.2. A larger lapse rate was allowed as researchers often cannot be certain what the true underlying lapse rate is (Wichmann & Hill, 2001; but see García-Pérez, 2014; Jones et al., 2015; Prins, 2012, 2013; Watson, 2017; Watson & Pelli, 1983; for alternative, adaptive estimation approaches). $F(x| \mu ,\sigma )$ describes the probability of responding that a comparison stimulus was bigger than a reference stimulus (which is typically of fixed size) as a function of the real comparison stimulus size *x*, modelled as cumulative Gaussian:

7 $$F(x| \mu ,\sigma )= \frac{1}{\sigma \sqrt{2\pi }}{\int }_{-\infty }^{x}{e}^{\frac{{-(t-\mu )}^{2}}{{2\sigma }^{2}}}dt$$

Here, $\mu$ refers to the mean of the cumulative Gaussian and describes the psychometric function’s point of subjective equivalence (e.g., stimulus size of comparison stimulus that is subjectively equivalent to the size of reference stimulus), while $\sigma$ refers to its standard deviation and links to the sensory noise of the cue.^6^

**Fig. 5:**
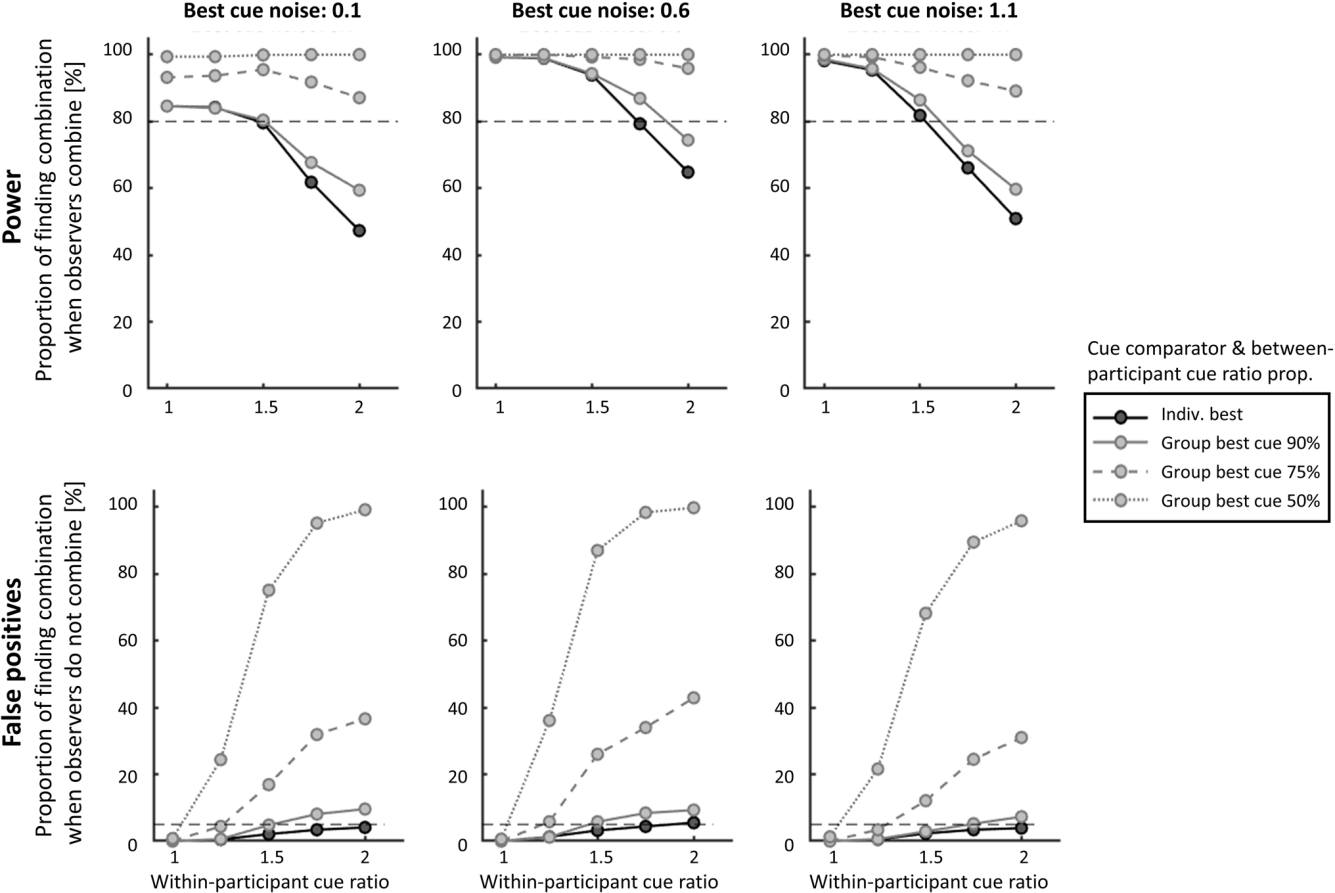
Each *point* represents the probability of finding significant cue combination effects in a number of simulated experiments (*n*_exp_ = 1000) in which observers (*n*_obs_ = 30) either combined the two cues according to statistically optimal predictions (power; *top panels*) or did not combine the cues but followed the single most reliable cue (false positives; *bottom panels*). Hence, the bottom row indicates the proportion of false combination effects*,* resulting from measurement noise and analysis approach. *Grey* and *black colours* indicate different analysis contrasts (Eq. 2, combined vs *group-determined best cue* and Eq. 3, combined vs *individually determined best cue*, respectively), while different *grey line types* show scenarios in which 50% (dotted), 75% (dashed) or 90% (*solid*) of participants show the same between-participant cue ratio, i.e., ${\sigma }_{1}$*<* ${\sigma }_{2}$. *Horizontal dashed lines in the upper panels* indicate 80% probability of detecting a combination effect, which can be interpreted as a quantification of power. An increase in sample size enhanced the chances of detecting combination effects (not shown here; but also see Scarfe, 2022). *Horizontal dashed lines in the lower panels* indicate the generally employed upper limit of tolerated alpha error of 5%

We simulated 1000 experiments, each consisting of 30 observers, which is leaning towards the higher end of sample sizes typically found in psychophysical cue combination experiments (Meijer et al., 2019; Rohde et al., 2016; Scheller et al., 2020). As outlined above, the probability of detecting cue combination in psychophysical experiments depends not only on design choices such as the sample size and analysis cue comparator, but also on further participant-specific characteristics such as lapses and the maximum possible benefit *B*_max_, that is, the best cue’s sensory noise level and the within-participant cue ratio. We therefore simulated all experiments for a range of plausible observer characteristics: observers differed in their best sensory noise levels between 0.1 and 1.1, with cue noise ratios between 1 (perfectly matched) and 2 (worse cue noise twice as high as best cue noise). These simulations were run for two scenarios: one scenario in which observers combined both cues optimally, and one in which observers followed the best sensory cue, i.e., did not combine the cues. For each of the resulting 30,000 simulated experiments (1000 experiments × 3 best sensory noise levels × 5 ratios × 2 combination scenarios) we applied the two different comparator contrasts: the combined condition was either compared with the *group-determined best cue* (Eq. 2; Fig. 5 grey points; see also Fig. 2a), or with the *individually determined best cue* (Eq. 3; Fig. 5 black points; see also Fig. 2b). In the former case, we further assumed that the between-participant cue ratio in the sample was either evenly split (50% ${\sigma }_{1}$*<* ${\sigma }_{2}$) or increasingly homogenous (75% ${\sigma }_{1}$*<* ${\sigma }_{2}$; 90% ${\sigma }_{1}$*<* ${\sigma }_{2}$), as this influences the degree of alpha error inflation. "Effects of the different cue contrasts" section shows that if all participants express the same relative cue ratio (100% ${\sigma }_{1}$*<* ${\sigma }_{2}$) the analysis does not differ from the combined vs *individually determined best cue* contrast, simply because the individually determined best cue is also the group’s best cue. As sensory noise values are typically not normally distributed, one-sided Wilcoxon signed-rank tests were used to test for significant decreases in sensory noise in the combined condition compared to the respective single cue condition. Figure 5 shows the proportion of experiments for which significant cue combination effects were found under the conditions that either all observers combined the cues according to statistically optimal predictions (100% combination probability) or no observer combined the cues (0% combination probability). Note that a within-participant cue ratio of 1 (equal cue reliabilities) presents the best-case scenario in which we can experimentally distinguish between combination and following the best single cue.

Comparing the effect of the two different analysis approaches (black and grey lines in Fig. 5), our simulations demonstrate that when observers do combine cues (top row), the probability of finding combination effects is larger when the combined cue condition is contrasted with the *group-determined best single cue* conditions (Eq. 2; grey points), compared to the *individually determined best single cue* (Eq. 3; black points). This, however, is also the case when the simulated observers do not combine (except in the special case of observers having exactly matched cue reliabilities – bottom left panel). In other words, even when observers do not combine cues but simply follow the more reliable cue, the former approach suggests that observers combine as a result of the single-cue noise inflation. This increase in falsely detecting combination effects greatly exceeds the generally accepted alpha level of 5% and is largest when cue between-participant cue ratio is most evenly split (50% ${\sigma }_{1}$*<* ${\sigma }_{2}$), with up to 100% of false positives. The proportion of false positives decreases as the same cue becomes more reliable across all participants (100% ${\sigma }_{1}$*<* ${\sigma }_{2}$) and when within-participant cue ratios become more matched. However, this incredibly high rate of false positives is alarming, given that the majority of published studies employed this type of analysis^1^. In comparison, the rate of false positives stays well within the 5% margin when an analysis is used that contrasts the combined condition with the *individually determined best cue* (Eq. 3).

Beyond the effect that the comparator choice has on the probability of finding true and false combination effects, our simulations show that the ability to distinguish true combination effects from alternative models decreases with increasing cue noise ratio and is highest when the individual cues reliabilities are well matched (cue ratio = 1; see also Scarfe, 2022). This is because the maximum achievable benefit (and hence the possible effect size) is largest when cues are matched. Furthermore, the probability of finding a combination is most pronounced within a certain range of sensory noise values, that is, for a normalized range between 0.2 and 1. This, again, can be explained by a combination of the maximum possible benefit in noise reduction that can be achieved (*B*_max_), as well as the enhanced conflation of sensory noise and measurement noise (e.g., lapse rate estimation) when uncertainty is high.

Note that the absolute probability of finding a true combination effect further depends on the sample size and precision (smallest possible measurement noise) that can be achieved by the study (Scarfe, 2022). An effect of measurement noise in the present simulations, for instance, is reflected in an increased inability to distinguish lapse rates from sensory noise when uncertainty is high. Furthermore, the statistically optimal cue combination model relies on assumptions that are not always tested by researchers (for more details, see Ernst, 2012; Rohde et al., 2016; Scarfe, 2022).

## Conclusion and best-practice suggestions

Studying how sensory information is integrated within or across multiple senses allows us to better understand perceptual computations that lie at the foundation of adaptive perception and behaviour. Specifically, the benefit in perceptual precision, accrued by combining the available sensory information in a statistically optimal fashion (Ernst & Banks, 2002), has received increasing attention, being termed nothing less than the “most important hallmark of optimal integration” (Rohde et al., 2016, p. 285). However, the precise quantification of perceptual precision that is often necessary to measure effects of such small sizes requires careful consideration. As has been demonstrated recently (Scarfe, 2022), many (influential) studies that report evidence for cue combination fall short on the ability to statistically test for such effects and distinguish between cue combination and alternative models, such as observers following the best sensory cue. While there are multiple participant-specific factors that cannot be determined in advance, such as the observer’s exact sensory noise ratio or the proportion of lapses observers will exhibit during a given session, careful study design and the correct choice of analysis are crucial to achieve maximum credibility of the reported effects.

Firstly, as cue combination necessarily leads to a benefit in perceptual precision when both cues are present, the crucial criterion that researchers should test for is a decrease in sensory noise (or increase in precision) in the combined cue condition compared to the best single-cue condition. Comparing the combined sensory noise levels against optimal predictions is not enough, as it does not evidence a perceptual precision benefit.

Importantly, adding to the design considerations outlined by Scarfe (2022), the present study demonstrates that the analysis used to test this criterion needs to be revisited, as it suffers from a large alpha error inflation. Specifically, here we demonstrated that the choice of cue comparator (*group-determined best single cue* or *individually determined best single cue*) has huge implications for whether a reported combination effect reflects *true combination*. Only contrasting the combined noise levels with the *individually determined best cue* allows to measure true cue combination. However, the majority of published cue combination studies^1^ to date contrasted the combined noise levels with the *group-determined best cue*. Here we showed that this method risks a strong inflation of false positives, with chances of falsely reporting cue combination as large as 100%. Notably, the studies that used this comparator were not only more common but also received more citations per year^1^ than the ones using the correct cue comparator, which may suggest that they were more influential.

The degree of false-positive inflation depends on several participant-specific characteristics: the within-participant cue ratio, the absolute sensory noise levels in the individual cues, as well as the between-participant cue ratio proportion (e.g., ~ 50% ${\sigma }_{1}$ < ${\sigma }_{2}$). If all participants show higher noise levels in the same cue, the analyses are equivalent. However, this is rarely the case in cue combination studies, especially when the cues are approximately matched, which is desirable to achieve larger possible effect sizes. Therefore, the approach involving the group-determined best (and worst) cue(s) as comparator is not recommended. Luckily, as researchers we have complete control over the comparator choice and implementing the correct comparison that allows us to maintain confidence that we are measuring a true combination effect merely requires one extra step. That is, out of the two individual cues, the best cue for each individual needs to be determined before contrasts are applied.

Based on the above demonstration, we outline several recommendations for researchers that study how sensory information is integrated using a cue combination approach:

1. Employ an analysis that minimizes the possibility of producing false combination effects. As *true combination* necessarily results in the decrease of sensory uncertainty in the combined cue condition, relative to the *individually determined best cue*, the choice of analysis needs to reflect this (Eq. 3).
2. Additionally, illustrating combination effects at the individual level is often useful, especially when it supplements group-level analyses. This provides an estimate of the overall prevalence and individual degree of combination effects within the group.
3. Testing whether the precision benefit follows (optimal) MLE predictions should be an additional, but not an alternative, step when aiming to evidence combination/integration of two cues. The degree of combination can also be quantified as difference between the minimal possible sensory noise and the empirically measured combined noise level (Eq. 5). This is because the MLE prediction provides the maximum possible benefit/minimum possible noise level that can be measured, taking the observer’s unisensory variances and variance ratio into account. As such, this combination index may be especially useful if a simple, quantified measure of integration degree (relative to what is maximally possible) is needed to contrast between groups. Note, however, that similar to the contrast with optimal predictions, this measure alone does not allow to infer whether integration took place, as it is still possible that participants followed the best single cue. To evidence combination, step 1 needs to be implemented.
4. Seconding previous recommendations (Ernst, 2012; Rohde et al., 2016; Scarfe, 2022), we remind researchers to carefully consider their design parameters in order to minimize measurement noise (e.g., maximize number of trial repetitions, select sensible stimulus levels and a suitable testing range that allows response proportions to plateau, select appropriate parameter estimation procedure and limits; Kingdom & Prins, 2016; Prins, 2012, 2013) and maximize power (e.g., define a sample size that takes the maximum benefit relative to the measurement noise into account, and maximize the possible benefit by matching single-cue noise levels; Rohde et al., 2016; Scarfe, 2022). Sensible stimulus presentation ranges and hardware-related measurement noise can be best determined in pilot studies. Furthermore, simulating data can be of great help to provide the researcher with an estimate of analysis-related measurement noise. Notably, the assumptions upon which cue combination models rest^7^ are often neglected, however their implications are vital for determining whether cue combination is present and whether it follows optimal predictions (Scarfe, 2022).

The implications that the comparator choice has on our ability to distinguish cue combination from alternative strategies is far reaching, and does not only affect planning of future studies, but also questions the results of published studies that have used the *group-determined best and worst cues* as comparators to evidence combination (this includes the authors’ own studies). Our recommendation therefore extends to researchers of published articles to re-analyse their data using the more appropriate comparator, that is, the *individually selected best cue*, to ascertain that their reported effects indeed reflect *true combination*.

Taken together, the present study advocates for a more careful comparator selection and task design in order to ensure cue combination is tested with maximum power while reducing the inflation of false positives. Clearly, while some factors that influence our ability to find true combination effects are more difficult to control or anticipate in advance, such as an observer’s absolute levels of sensory noise for a given cue, their sensory noise ratio, or expectable lapse rates^8^, the choice of analysis is a design factor that is under full researcher control.

### Supplementary Information

Below is the link to the electronic supplementary material.

Supplementary file1 (DOCX 336 KB)

## Data Availability

See Open Practices.

## Footnotes

1 Out of 45 studies that we screened, published between 2002 and 2022 (see "Different approaches to quantifying cue combination" section), 80% employed this error-prone analysis to test for cue combination. Furthermore, these studies were, on average, published in higher-impact-factor journals (average ± CI^95%^: 4.8 ± 1 vs. 3.4 ± 0.8) and received more citations per year (average ± CI^95%^: 10.7 ± 3.1 vs 6.2 ± 4.1; note that two very highly cited papers, Ernst & Banks, 2002, and Alais & Burr, 2004, are not included in these numbers). This is problematic, as it suggests that some of the more influential evidence is grounded on an error-prone analysis. Furthermore, it suggests that these wrong analysis choices are likely to perpetuate throughout the literature.

2 Note that measurement noise arising from parameter estimation and design parameters such as stimulus spacing and stimulus repetitions (Prins, 2012) affects sensory noise estimates across all conditions, affording the possibility of an underestimation (leading to apparent supra-optimal performance) or overestimation (apparent sub-optimal performance) of the true maximum possible benefit.

3 Studies that employed individual-level analyses typically aimed to enhance power by minimizing measurement error (for instance, by including a large number of trials per condition or testing multiple levels of noise and conflict in each participant). This typically requires participants to return for multiple sessions and limits the feasibility to test a large number of participants (trade-off between measurement precision and sample size).

4 These studies typically used a measure of precision to quantify cue combination; however, similar methods have been employed to evidence multisensory benefits through accuracy (or signal detection) and response time measures (e.g. Collignon et al., 2008; Denervaud et al., 2020; Girard et al., 2011; Heffer et al., 2022; Murray et al., 2018; Petrini et al., 2010).

5 As the theory-derived statistical optimality model provides point predictions (i.e. a quantified estimation of the expected benefit), individual-level analyses in small samples can be sufficiently meaningful to draw some conclusions about optimality of cue combination. However, there are a number of limitations associated with this approach beyond the reduced generalizability of the findings. For instance, both the empirically determined combined cue noise and the optimal point prediction, which is based on the empirically determined single-cue noise levels, remains affected by measurement noise. Hence, deviations from the point prediction can be expected simply based on measurement variability. Inferring whether the magnitude of deviation from point predictions arises from measurement noise or sub-optimality of the perceptual process is therefore often not possible. Nevertheless, while the focus of the present paper lies on the group-based analysis of combination effects, which has been most frequently employed, individual-based analyses that adopt a statistical (e.g. bootstrap) approach remain a viable alternative.

6 Note that, a cue's sensory noise (σ) relates to the standard deviation of the psychometric function via ${\sigma} = \sqrt{\frac{sd^2}{2}}$, that is, it relates to half of the variance.

7 Absence of perceptual bias (Scarfe & Hibbard, 2011) and learning effects throughout the task (Fründ et al., 2011); reduced decisional noise (Hillis et al., 2004); Independence of sensory noise (Oruç et al., 2003)

8 It is still possible to get an idea of the to be expected parameters. Rigorous piloting, as well as adjustment of the stimulus range to the individual noise levels offer possibilities to gain better control over these parameters (Rohde et al., 2016; Meijer et al., 2019). However, precise noise level estimation is typically time intensive and requires many trial repetitions for each cue. This may require researchers to plan additional experimental sessions for stimulus adjustments, which is not always feasible. Also, as there is individual variability across days (e.g. if two cues are matched on one day, there may be a slight mismatch on another day depending on participant-specific characteristic and circumstances) and residual measurement noise in the parameter estimation procedure, the exact matching of cues is rarely possible. However, these options allow to keep the within-participant cue ratio to a minimum and provide the best basis for testing for true cue combination effects.
