# Supplementary material for: Correctly establishing evidence for cue combination via gains in sensory precision: Why the choice of comparator matters
Source: Behav Res Methods. 2023 Sep 20;56(4):2842–58. doi: 10.3758/s13428-023-02227-w (PMC11133123; doi:10.3758/s13428-023-02227-w)
Supplement: Supplementary file 1 — Supplementary file1 (DOCX 336 KB) [file 13428_2023_2227_MOESM1_ESM.docx]

**Supplementary information**

Estimating parameters of perceptual precision (such as the psychometric function slope) becomes increasingly uncertain as sensory noise increases (i.e., stimulus discriminability reduces). One can think of selecting a narrow stimulus range, within which discriminating two stimuli is difficult, resulting in a shallow psychometric function. Especially when additional parameters, such as the lapse rate, which is typically unknown to the experimenter, is estimated alongside parameters of interest (slope) the estimation uncertainty increases. This is because it is unclear whether the variability in responses at the extreme ends of the range results from reduced perceptual precision (small slope) or from an increase in attentional lapses.

Notably, while the data-driven estimation of nuisance parameters such as the lapse rate is debated (Prins, 2012; Treutwein, 1999; Wichmann & Hill, 2001), grossly over- or underestimating this parameter will almost certainly lead to biases in the parameter estimates of interest (sensory noise). To illustrate this example, we ran simulations in which observers with different sensory noise levels and different lapse rates were generated. Sensory noise values were randomly drawn from a truncated normal distribution centred on values between 0.1 and 1.1 (SD = 0.05). Lapse rates were systematically varied between 1% and 20% (the latter being less likely, but not impossible) to assess their influence on sensory noise parameter recoverability. Their data was then fit with psychometric functions to estimate their sensory noise parameters. For each case, we ran 1000 simulations, each of which generated 35 observers across which recoverability parameters (correlation coefficient r and median bias) were measured. We further varied the range of possible lapse rate values that our parameter estimation procedure allowed to fit (i.e., lapse rate constrain). These simulations showed that, firstly, larger sensory noise values were less well recovered than lower sensory noise values (see Figure S1). In other words, the less precise the cue, the less reliably could it be recovered. Secondly, unsurprisingly, the larger the lapse rate the more difficult it was to recover the simulated sensory noise parameters. Thirdly, median bias between the simulated and estimated sensory noise levels increased, depending on the sensory noise value (higher noise values = larger bias). The directionality and degree of this bias further depends on the limits within which the lapse rate is allowed to vary. Across all cases, parameter estimation was more reliable (higher recoverability) and less biased when the underlying psychometric function was steeper, i.e., if it plateaued at the extremes. Furthermore, even constraining the lapse rate to vary within a limited range can induce bias in the estimation of sensory noise parameters. Hence, researchers need to decide whether they constrain or fix the lapse rate, keeping possible bias in mind, or estimate lapse rates in psychophysical tasks (Prins, 2012; Treutwein, 1999; Wichmann & Hill, 2001). In either case, with increasing uncertainty, lapses and sensory noise becomes less distinguishable from each other, which would argue against increasing the sensory noise in the best single cue, even if maximum possible benefits are comparably large. Instead, it argues for matching cue reliabilities in the individual cues as much as possible.

*
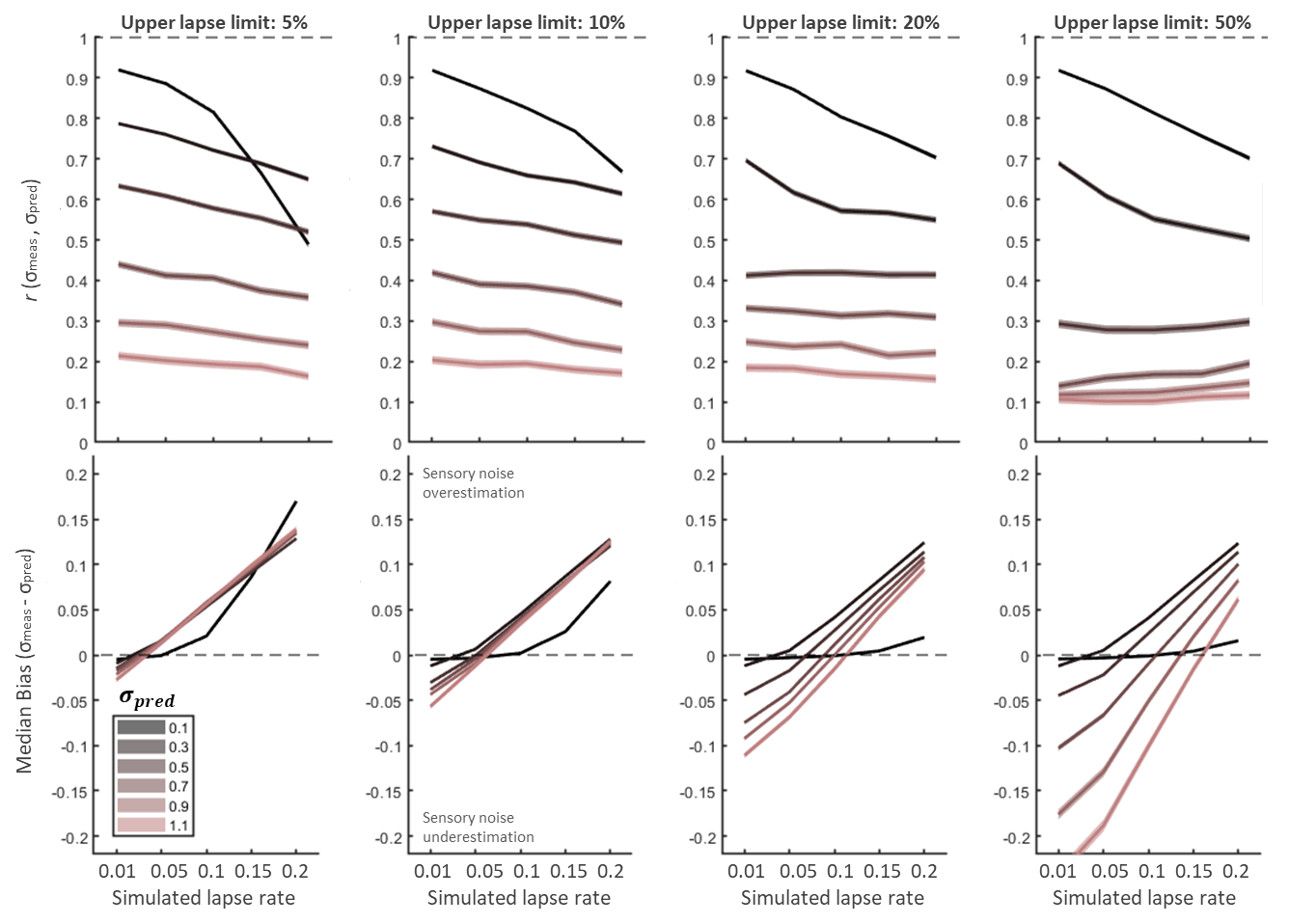
***Figure S1:** Sensory noise level recovery parameters for different simulated lapse rates and lapse estimation limits. Each figure shows how well different noise levels can be recovered depending on the degree of lapses (between 1-20% of trials). Correlation coefficient *r* (upper row) and the median bias (lower row) were measured for a set of simulated *(*$\sigma_{pred})$ and recovered *(*$\sigma_{meas})$ noise levels across 1000 experiments with 35 observers each. Simulated sensory noise levels were drawn randomly from a truncated normal distribution centred on values between 0.1 and 1.1 (SD = 0.05). Shaded bands indicate 95% confidence intervals. Different panels in each row indicate the correlation and median bias when different levels of lapses are allowed in the fitting procedure. Lower simulated sensory noise values show higher recoverability, while increasing sensory noise levels are more often conflated with estimated lapse rates, depending both on the degree of lapses as well as the maximum degree of lapses allowed in the fitting procedure. The effect of absolute sensory noise value increases with increasing fitting limits.

**References**

Prins, N. (2012). The psychometric function: The lapse rate revisited. *Journal of Vision*, *12*(6). https://doi.org/10.1167/12.6.25

Treutwein, B. (1999). Fitting the psychometric function. *Perception & Psychophysics*, *61*(1), 87–106.

Wichmann, F. A., & Hill, N. J. (2001). The psychometric function: I. Fitting, sampling, and goodness of fit. *Perception & Psychophysics 2001 63:8*, *63*(8), 1293–1313. https://doi.org/10.3758/BF03194544
